# The increasing importance of histologic grading in tailoring adjuvant systemic therapy in 30,843 breast cancer patients

**DOI:** 10.1007/s10549-021-06098-7

**Published:** 2021-01-30

**Authors:** C. van Dooijeweert, I. O. Baas, I. A. G. Deckers, S. Siesling, P. J. van Diest, E. van der Wall

**Affiliations:** 1 Department of Pathology, University Medical Center Utrecht, PO Box 85500, 3508 GA Utrecht, The Netherlands; 2 Department of Medical Oncology, University Medical Center Utrecht, Utrecht, The Netherlands; 3 Foundation PALGA (the Nationwide Network and Registry of Histo- and Cytopathology in the Netherlands), Houten, The Netherlands; 4 Department of Research, Netherlands Comprehensive Cancer Organization, Utrecht, The Netherlands; 5 Department of Health Technology & Services Research, Technical Medical Centre, University of Twente, Enschede, The Netherlands

**Keywords:** Invasive breast cancer, Histologic grade, Guideline adherence, Adjuvant systemic treatment, Real-world data

## Abstract

**Purpose:**

The large variation in histologic grading of invasive breast cancer (IBC) that has been reported likely influences tailoring adjuvant therapy. The role of grading in therapeutic decision-making in daily practice, was evaluated using the Dutch national guidelines for IBC-management.

**Methods:**

Synoptic reports of IBC resection-specimens, obtained between 2013 and 2016, were extracted from the nationwide Dutch Pathology Registry, and linked to treatment-data from the Netherlands Cancer Registry. The relevance of grading for adjuvant chemotherapy (aCT) was quantified by identifying patients for whom grade was the determinative factor. In addition, the relation between grade and aCT-administration was evaluated by multivariate logistic regression for patients with a guideline-aCT-indication.

**Results:**

30,843 patients were included. Applying the guideline that was valid between 2013 and 2016, grade was the determinative factor for the aCT-indication in 7744 (25.1%) patients, a percentage that even increased according to the current guideline where grade would be decisive for aCT in 10,869 (35.2%) patients. Also in current practice, the indication for adjuvant endocrine therapy (aET) would be based on grade in 9173 (29.7%) patients. Finally, as patients with lower-grade tumors receive aCT significantly less often, grade was also decisive in tailoring aCT de-escalation.

**Conclusions:**

In the largest study published so far we illustrate the increasing importance of histologic grade in tailoring adjuvant systemic breast cancer therapy. Next to playing a key-role in aCT-indication and de-escalation, the role of grading has expanded to the indication for aET. Optimizing histologic grading by pathologists is urgently needed to diminish the risk of worse patient outcome due to non-optimal treatment.

**Supplementary Information:**

The online version contains supplementary material available at 10.1007/s10549-021-06098-7.

## Introduction

Breast cancer is the most common type of cancer in women worldwide, with an incidence of 2.1 million [1]. In the Netherlands, invasive breast cancer (IBC) accounts for approximately 15,000 new diagnoses annually [2, 3].

Aiming to provide individual IBC-management, treatment is guided by clinicopathologic biomarkers [4–12]. For example, the indication for adjuvant systemic chemotherapy (aCT), is based on patient factors like age and performance status, and classic pathology features like tumor size, lymph-node status, grade, and estrogen- (ER), progesterone- (PR), and HER2-receptor status [5–9, 11, 13, 14]. Of these, histologic grade, according to the (globally used) Bloom and Richardson grading classification, is a biomarker that has consistently been found to be associated with breast cancer-specific and disease free-survival [13, 14].

Within the Dutch IBC guideline, in line with global breast cancer guidelines [4–10], grade plays an important role in the selection of individual patients considered to experience benefit from aCT [11]. We have previously shown that substantial grading variation exists between Dutch pathology laboratories in daily practice [15], which makes it highly likely that tumors are under- and over-graded in specific pathology laboratories and/or by specific pathologists. In view of its decisive role in personalized treatment, this variation is worrisome to the least since it may lead to under- or over-treatment.

In selecting the right patients for adjuvant systemic chemotherapy (aCT), most guidelines roughly distinguish three groups, i.e. estrogen (ER +)- and or progesterone (PR +)-driven HER2-negative IBC, HER2-driven (ER-/PR-, HER2 +) IBC and triple negative IBC (TNBC) (ER-, PR-, HER2-) [4, 6, 7, 10–12]. For HER2-driven IBC and TNBC, these guidelines state that practically all patients should receive aCT [6, 7, 10–12]. In contrast, for ER and/or PR-driven IBC, focus has shifted to tailored aCT de-escalation, thereby mostly maintaining adjuvant endocrine therapy (aET).

More recently, gene-expression profiling (GEP) by multigene-assays (i.e. the 21-gene recurrence score assay (21-GS/Oncotype DX) and the 70-gene signature (70-GS/MammaPrint) has been introduced as a supportive tool in clinical-decision making for ER + /HER2− IBC [5–9, 11, 13, 14]. The Dutch breast cancer guideline [11] encourages the 70-GS in in those patients that have tumors characterized by being ER + , HER2−, having stage pT2N0 or pT1N1 (1 lymph-node) and being of ductal/no special type (NST). The GEP-indication further depends on tumor size and also on grade [11].

Histologic grade clearly plays an important role in tailoring aCT, both directly and indirectly. Considering our earlier data on the existence of substantial variation in grading in daily pathology practice, we decided to evaluate the role of grading in tailoring adjuvant systemic therapy in a nationwide setting, taking both the past and current Dutch IBC-guideline into consideration.

## Material and methods

### Key objectives

The key-objectives of this study were to evaluate the importance of grading in tailoring adjuvant systemic therapy within the past and current Dutch breast cancer guideline, by identifying specific groups of patients for whom the systemic therapy indication depends on histologic grade. Second, we evaluated guideline adherence to the past Dutch breast cancer guideline, and we evaluated which factors, among which histologic grade, may have influenced this.

### Study population

Data were primarily extracted from the nationwide Dutch Pathology Registry (PALGA). All synoptic pathology reports of IBC resection-specimens in the Netherlands, obtained between January 1, 2013, and December 1, 2016, were extracted from PALGA, including *n* = 48,667 reports from 42,705 patients. From each report we extracted patient- (sex, age, neoadjuvant treatment) and tumor characteristics (size, grade, histologic subtype, and ER-, PR- and HER2-status).

Reports of patients without a primary tumor (*n* = 2104) were excluded. Furthermore, reports from patients who received neoadjuvant treatment (*n* = 5829) were excluded, because treatment for these patients is based upon biopsies, whereas biomarkers like grade, and receptor-status may alter after systemic treatment [16–21], and as such may differ from the initial biopsy. For synchronous IBC, defined as ipsilateral IBC within six months after the first IBC resection-specimen report, only the first report was included, as this tumor is usually the index-tumor on which treatment decisions are based. Remaining patients with > 1 pathology report (*n* = 1495), which concerned bilateral tumors in the majority (> 90%), were excluded, as it could not be determined which tumor was considered the index-tumor. Lastly, we excluded reports with any missing data (which concerned missing values on grade, ER/PR-, and HER2-receptor status) (eFigure 1).

To evaluate the role of grading in tailoring adjuvant systemic therapy in daily practice, the pathology-specific data were linked to treatment data from the Netherlands Cancer Registry (NCR) adding information on adjuvant systemic treatment and information on N-status. In the final step of linking, < 5% of patients (1454) could not be matched, leaving a total of 30,843 IBC patients for data analysis (eFigure 1).

This study was approved by the scientific and privacy committee of PALGA and the monitory board of the NCR.

### Histologic grade and other determinants

Grade was assessed according to the modified Bloom and Richardson guideline, which combines three sub-scores (nuclear pleomorphism, tubule formation and mitotic count), resulting in a total score and derived grade [22, 23]. The combined ER/PR receptor-status was considered positive when ≥ 10% of tumor cells showed nuclear staining by immunohistochemistry (IHC) for either or both ER and PR, which is the cutoff used in the Netherlands [12]. Based on guideline cutoffs, tumor size was categorized (≤ 1 cm, 1.1-2 cm, > 2 cm). For age, we used different cutoffs based on the guideline indication, as this was deemed clinically most relevant. For GEPs, we only presented numbers on 70-GS, as 21-GS was barely used in the Netherlands between 2013 and 2016 (< 1%) [24].

### Adjuvant systemic therapy and grading within the past and present guideline

According to the guideline that was valid during the study period (2013–2016) [25], four patient groups had an aCT-indication. First this included all N + patients < 70 years (subgroup 1). Second this concerned N0 patients < 70 years with unfavorable characteristics, which were defined as age < 35 years (except grade I tumors ≤ 1 cm) (subgroup 2), or, within patients aged 35–70, grade II-III tumors of 1.1-2 cm (subgroup 3), and all tumors > 2 cm (subgroup 4).

When specifically focusing on grade, it can be concluded that the aCT-indication according to the 2013–2016 guideline specifically depended on grade (i.e. grade was the determinative factor) in patients < 35 years with a N0 tumor ≤ 1 cm, and in patients aged 35–70 with a N0 tumor between 1.1 and 2 cm, as aCT was indicated when their tumor was graded as ≥ grade II.

To evaluate whether accurate grading by pathologists remains relevant, we then evaluated grading within the current Dutch breast cancer guideline, which was published in 2019 [11]. Currently, aCT is indicated in N + patients < 70 years (except grade I tumors < 2 cm) (subgroup 1), and patients < 70 years N0/N0(i +)/N1(mi) with unfavorable characteristics. These unfavorable characteristics are defined as age < 35 years with a grade I tumor > 2 cm, or a grade II/III tumor > 1 cm (subgroup 2), age 35–70 with a grade I tumor > 3 cm, a grade II tumor > 2 cm, or a grade III tumor > 1 cm (subgroup 3). Lastly, aCT is indicated in all HER2-overexpressing tumors (subgroup 4).

Thus the aCT indication within the current guideline depends on grade in three specific patient subgroups. First this concerns patients < 35 years, N0/N0(i +)/N1(mi) with a HER2-negative tumor between 1.1 and 2 cm. Second, this concerns patients aged 35–70, N1-3, and a hormone-positive, HER2-negative tumor ≤ 2 cm. Lastly, this concerns patients aged 35–70, N0/N0(i +)/N1(mi) with a hormone receptor positive, HER2-negative tumor between 1.1 and 3 cm [11].

In addition, within the current guideline, grading also plays a role in tailoring (aET). Overall, aET is indicated in patients of all ages with an ER- and/or PR-positive tumor receptor status and N1-status (including (N1(mi)), while in case of an N0-status (including N0(i +)), aET is only indicated in grade I tumors > 2 cm, and ≥ grade II tumors > 1 cm. Thus, for N0/N0(i +) patients with a hormone-positive tumor between 1.1 and 2 cm, the aET-indication is determined by grade.

### Statistical analysis

Patient-, tumor- and treatment characteristics were summarized using counts and proportions, and means and standard deviations.

First, we identified patients for whom the aCT-indication, when strictly applying the 2013–2016 guideline, depended on grade. We then performed the same analyses for the 2019 guideline, and we also identified patients for whom the aET-indication would depend on grade, as this was added in the 2019 guideline. Second, we calculated which proportion of patients with an aCT-indication according to the 2013–2016 guideline actually received aCT. Third, the relation of grade and other clinicopathologic variables (age, ER/PR- and HER2-receptor status, tumor size) and 70-GS use with aCT-administration was evaluated by multivariate logistic regression for the overall population with a 2013–2016 guideline aCT-indication (i.e. all four subgroups). Adjusted odds ratios (AORs) and 95% confidence intervals (CIs) were provided for aCT versus no aCT. Grade I was taken into account as reference category for grade. Lastly, to identify factors that may have influenced guideline adherence with regard to aCT, within the four subgroups with a guideline aCT-indication, we compared clinicopathologic variables (grade, ER/PR- and HER2-receptor status, age, tumor size) and 70-GS-use between patients who did and did not receive aCT, using counts and proportions, which were tested by means of a *χ*^2^-test. For age, a cutoff of ≥ 60 years was used, as a considerable group of patients with an aCT indication was aged between 60 and 70 years, which may have influenced guideline adherence.

*p*-values < 0.05 were considered statistically significant. All statistical analyses were performed by using IBM SPSS Statistics version 15.0.0.2.

## Results

Characteristics of the 30,843 included patients are summarized in Table 1. The overall mean age was 62.0 years, while about a quarter of patients were ≥ 70 years. Only 245 males were included (0.8%). Patients were N0 in roughly two thirds of cases (68.6%) and a similar percentage of patients underwent breast conserving surgery (66.1%). Mean tumor size was just under 2 cm and the majority of tumors (84.7%) were of ductal/no special type (NST) subtype. Hormone-receptor positivity was reported in 87.6% of tumors and 10.0% were HER2-receptor positive.

**Table 1:** Characteristics of a Dutch nationwide cohort of breast cancer patients (*n* = 30,843), without neo-adjuvant treatment, with a single synoptic resection- specimen pathology report between 2013 and 2016

Characteristics	Total (*N* = 30,843)
Age (years) [mean (SD)]	62.0 (12.0)
< 60 years	12,572 (40.8%)
≥ 60 years	18,271 (59.2%)
< 70 years	22,338 (72.4%)
≥ 70 years	8505 (27.6%)
Sex [*n* (%)]	
Female	30,598 (99.2%)
Male	245 (0.8%)
Lymph-node status [*n* (%)]	
N0	21,148 (68.6%)
N1	7295 (23.7%)
N2	996 (3.2%)
N3	606 (2.0%)
N unknown	798 (2.6%)
Type of surgery [*n* (%)]	
Mastectomy	10,463 (33.9%)
Breast conserving	20,380 (66.1%)
Tumor size (cm) [mean (SD)]	1.9 (1.3)
T-stage	
T1	21,393 (69.4%)
T2	8383 (27.2%)
T3	819 (2.7%)
T4	248 (0.8%)
Histologic subtype [*n* (%)]	
Ductal	26,121 (84.7%)
Lobular	3982 (12.9%)
Other	740 (2.4%)
Histologic grade [*n* (%)]	
Grade I	8633 (28.0%)
Grade II	14,682 (47.6%)
Grade III	7528 (24.4%)
ER/PR receptor status [*n* (%)]	
Positive	27,008 (87.6%)
Negative	3835 (12.4%)
HER2-receptor status [*n* (%)]	
Positive	3070 (10.0%)
Negative	27,773 (90.0%)
Triple negative status [*n* (%)]	2884 (9.4%)
Chemotherapy [*n* (%)]	9950 (32.3%)
Endocrine therapy [*n* (%)]	17,041 (55.3%)
Radiotherapy [*n* (%)]	22,186 (71.9%)
Targeted therapy [*n* (%)]	2281 (7.4%)
70-GS [*n* (%)]	3291 (10.7%)

Overall, aCT was administered in nearly one third of patients (32.3%), more than half of all patients received aET (55.3%), and 71.9% underwent radiotherapy. Targeted therapy was administered in 7.4% of patients, and consisted of the anti-HER2 agent Trastuzumab in nearly all cases. Little over ten percent of patients had a GEP performed, which concerned 70-GS in virtually all cases. The majority of patients with a 70-GS performed were assigned to the 70-GS low-risk category (*n* = 2026, 61.6%).

### Grading

We identified 7744 (25.1%) patients for whom grade would be the determinative factor for their aCT-indication, when strictly applying the guideline that was valid during the study period (2013–2016) (eTable 1). When applying the guideline that was published in 2019 [11], we identified an increase in the percentage of patients for whom the aCT-indication would depend on grade, being 35.2% (*n* = 10,869) (eTable 2). In addition, new in the current guideline, the indication for aET would depend on grade in 9,173 (29.7%) patients (eTable 3).

**Fig. 1:**
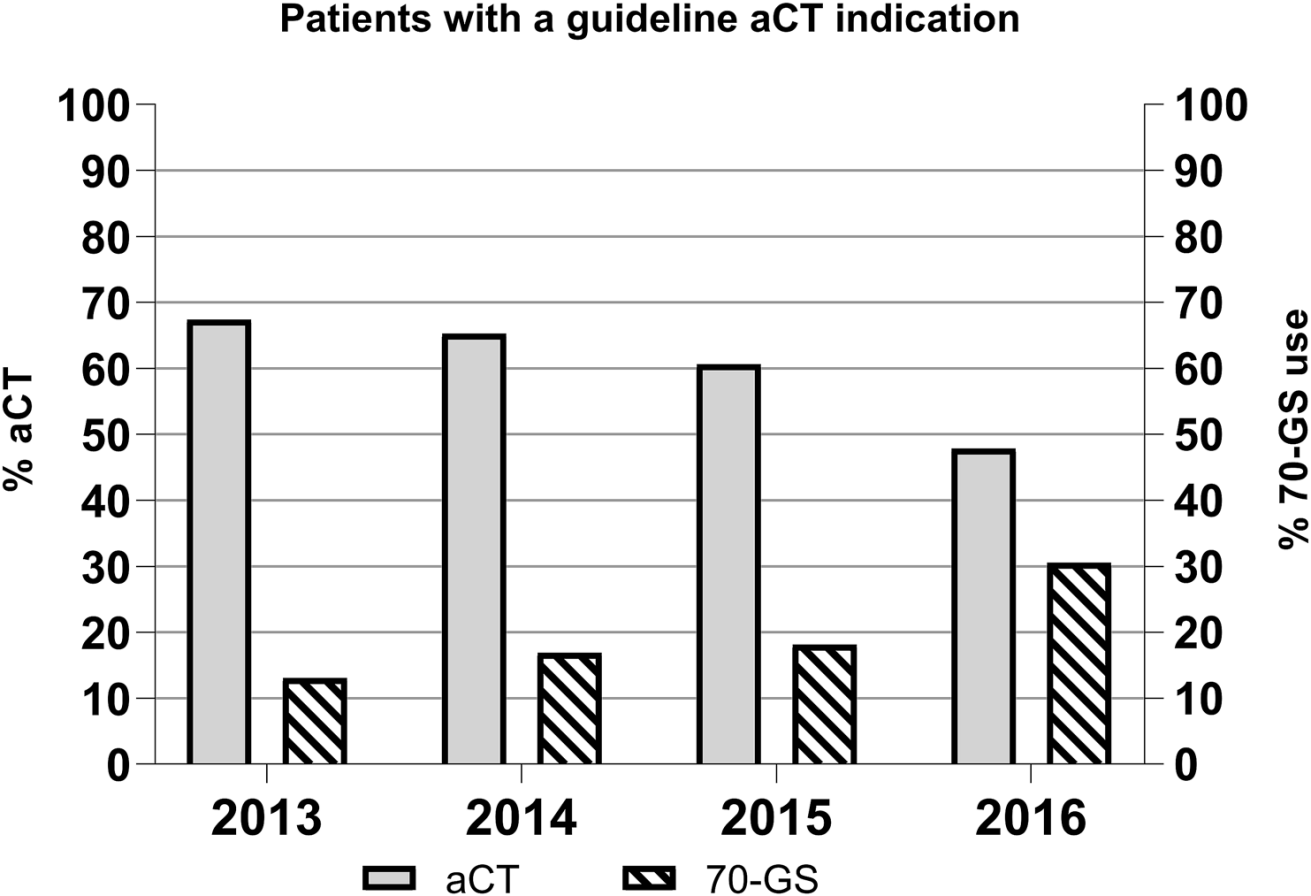
Patients with a strict aCT guideline indication according to the 2013–2016 guideline

Applying the Dutch breast cancer guideline that was valid between 2013 and 2016, we identified 14,954 patients (48.5%) with an aCT-indication (Table 2). Of this group, only 9,010 patients (60.3%) actually received aCT. As 9,950 patients actually received aCT, 940 patients (9.4%) apparently did so without having a guideline-indication. Administration of aCT decreased slightly between 2013 and 2015, while a considerable decrease of 12.8% was observed in 2016 (Fig. 1).

**Table 2:** Number of breast cancer patients from our dataset (2013–2016) with an indication for adjuvant chemotherapy (aCT) according to the Dutch breast cancer guideline between 2013 and 2016 vs. number of breast cancer patients who actually received aCT

Characteristics	Number of patients with an indication for aCT	Number of patients who received aCT
All patients < 70 years and N +	6378	4562
Patients up to 70 years and N0		
Age < 35 years: grade I tumors > 1 cm, grade II–III all tumor sizes	202	180
Age ≥ 35 years: tumor 1.1–2 cm, grade II–III tumor	5459	2476
Age ≥ 35: tumor > 2 cm	2915	1792
All other patients	–	940
Total number of patients (*n* = 30.843)	14,954 (48.5%)	9950 (32.3%)

In general, for all patients with an aCT-indication, aCT-administration was significantly more apparent in patients with tumors of higher grade [adjusted OR (AOR) grade II: 1.4 (95%CI 1.3–1.7), AOR grade III: 4.7 (95%CI 4.1–5.4)], HER2-receptor-positive tumors [AOR 4.9 (95%CI 4.1–5.8)], and tumors of larger size [AOR per cm increase: 1.4 (95%CI 1.3–1.4)]. In contrast, patients with hormone-receptor-positive tumors (AOR: 0.4 (95%CI 0.4–0.5), an age ≥ 60 years (AOR: 0.3 (95% CI 0.3–0.3), or patients with a 70-GS performed (irrespective of 70-GS result-category) (AOR: 0.4 (95%CI 0.4–0.4) had significantly lower odds of receiving aCT (Table 3).

**Table 3:** Indicators of guideline adherence (aCT-administration) in 14,954 IBC patients with a guideline indication for adjuvant chemotherapy (aCT) according to the Dutch guideline (2013–2016)

Patient- and tumor characteristics	Number of patients	Adjusted OR (95% CI)*
Histologic grade		
Grade I	1732 (11.6%)	1
Grade II	8333 (55.7%)	1.5 (1.3–1.7)
Grade III	4889 (32.7%)	4.7 (4.1–5.4)
ER/PR positive receptor status	12,704 (85.0%)	0.4 (0.4–0.5)
HER2 positive receptor status	1921 (12.8%)	4.9 (4.1–5.8)
Age ≥ 60 years	6084 (40.7%)	0.3 (0.3–0.3)
Tumor size (cm)	2.1 (1.3)**	1.4 (1.3–1.4)
70-GS	2950 (19.7%)	0.4 (0.4–0.4)

*Calculated by multivariate logistic regression; adjusted for ER/PR-receptor status, HER2-receptor status, histologic grade, age, tumor size and 70-GS use

**Mean (SD)

Guideline adherence was most apparent for patients < 35 years with an aCT-indication (89.1%), while it was lowest for the group of N0-patients aged 35–69 with tumors of 1.1-2 cm size (45.4%) (Table 4). Notably, within the latter group, only 20.9% of patients with grade II tumors received aCT, whereas 78.8% of patients with grade III tumors received aCT *(p* = 0.000). In addition, 70-GS-use was highest within this group (30.2%) and aCT was only administered in 34.6% of these patients.

**Table 4:** Characteristics of IBC patients with an indication for adjuvant chemotherapy (aCT) according to the Dutch guideline (2013–2016) who received aCT versus patients with an aCT indication who did not receive aCT

Characteristics	Total	aCT received	No aCT received	*p*-value*
Patients < 70 years, N + tumor	6378	4562 (71.5%)	1816 (28.5%)	**0.000**
Histologic grade				
Grade I	1316	628 (477%)	688 (52.3%)	**0.000**
Grade II	3245	2363 (72.8%)	882 (27.2%)	
Grade III	1817	1571 (86.5%)	246 (13.5%)	
Receptor status				
ER and/or PR positive	5657	3899 (68.9%)	1758 (31.1%)	**0.000**
HER2 positive	858	794 (92.5%)	64 (7.5%)	**0.000**
Age ≥ 60 years	2403	1394 (58.0%)	1009 (42.0%)	**0.000**
Tumor size				
Tumor size ≤ 1.0 cm	623	321 (51.5%)	302 (48.5%)	**0.000**
Tumor size 1.1–2 cm	2826	1869 (66.1%)	957 (33.9%)	
Tumor size > 2 cm	2929	2372 (81.0%)	557 (19.0%)	
70-GS	721	240 (33.3%)	481 (66.7%)	**0.000**
Patients < 35 years, N0 tumor: grade I > 1 cm, grade II–III	202	180 (89.1%)	22 (10.9%)	**0.000**
Histologic grade				
Grade I	12	9 (75.0%)	3 (25.0%)	**0.000**
Grade II	61	46 (75.4%)	15 (24.6%)	
Grade III	129	125 (96.9%)	4 (3.1%)	
Receptor status				
ER and/or PR positive	112	93 (83.0%)	19 (17.0%)	**0.002**
HER2 positive	43	40 (93.0%)	3 (7.0%)	0.353
Tumor size				
Tumor size ≤ 1.0 cm	37	26 (70.3%)	11 (29.7%)	**0.000**
Tumor size 1.1–2 cm	99	91 (91.9%)	8 (8.1%)	
Tumor size > 2 cm	66	63 (95.5%)	3 (4.5%)	
70-GS	11	3 (27.3%)	8 (72.7%)	0.073
Patients 35–69 years, N0 tumor: 1.1–2 cm, grade II–III	5459	2476 (45.4%)	2,983 (54.6%)	**0.000**
Histologic grade				
Grade II	3686	1078 (29.2%)	2608 (70.8%)	**0.000**
Grade III	1773	1398 (78.8%)	375 (21.2%)	
Receptor status				
ER and/or PR positive	4636	1782 (38.4%)	2854 (61.6%)	**0.000**
HER2 positive	690	622 (90.1%)	68 (9.9%)	**0.000**
Age ≥ 60 years	2442	774 (31.7%)	1668 (68.3%)	**0.000**
70-GS	1648	571 (34.6%)	1077 (65.4%)	**0.000**
Patients 35–69 years, N0 tumor: > 2 cm	2915	1792 (61.5%)	1123 (38.5%)	**0.000**
Histologic grade				
Grade I	404	83 (20.5%)	321 (79.5%)	**0.000**
Grade II	1341	723 (53.9%)	618 (46.1%)	
Grade III	1170	986 (84.3%)	184 (15.7%)	
Receptor status				
ER and/or PR positive	2299	1248 (56.0%)	1051 (47.2%)	**0.000**
HER2 positive	330	294 (89.1%)	36 (10.9%)	**0.000**
Age ≥ 60 years	1239	578 (46.7%)	661 (53.3%)	**0.000**
70-GS	570	204 (35.8%)	366 (64.2%)	**0.000**

*Calculated by means of a χ^2^ test, bold numbers indicate statistical significance

As to the role of the 70-GS, this was used in 2,950 patients with a guideline-aCT-indication (19.7%), and only about a third of these patients received aCT (Table 4). We also observed an only marginal increase in the use of 70-GS in the years between 2013 and 2015, followed by a considerable increase of 12.4% in 2016 (Fig. 1).

## Discussion

Using real-world nationwide data in 30,843 IBC patients, the role of grading in daily practice, in light of both the past and present Dutch breast cancer guidelines, was evaluated.

These data illustrate that grade remains a key-player in tailoring adjuvant systemic therapy, and that the importance of accurate grading has only increased for aCT, as well as aET. As to the role of grading within the guideline that was valid during the study years, our data illustrate that grade played a significant role in tailoring aCT. Whereas the aCT-indication theoretically depended on grade in a quarter of IBC patients, grade also played an important role in tailoring aCT de-escalation, as patients with lower grade tumors received aCT significantly less often.

In general, these data illustrate a trend of aCT de-escalation in IBC patients whose risk of distant metastases was deemed low enough to withhold adjuvant systemic treatment. Important supportive clinicopathologic biomarkers tailoring aCT were patients’ age, N status, hormone- and HER2-receptor status, tumor size and grade. A particularly useful tool in aCT de-escalation in the Netherlands has been the 70-GS, whose use increased considerably in 2016, most likely after publication of the MINDACT-trial [26]. In patients with a strict guideline aCT-indication, this increase in 70-GS-use was accompanied by a decrease of aCT-administration. Overall, only a third of patients who had a 70-GS performed, received aCT. It is however important to emphasize that the observed aCT de-escalation can only partially be contributed to 70-GS-use, since, overall, 5944 patients with a strict guideline aCT-indication (40.7%) did not receive aCT. Of these patients, only 1932 (32.5%) had a 70-GS test, leaving 4012 (67.5%) patients, whose guideline non-adherence was probably based on clinicopathologic biomarkers. In addition, guideline (non)adherence may have also been influenced by other patient factors like comorbidity or patient preference. Although we did not have these data, patients of older age were less likely to receive aCT, which may have been related to existing comorbidities.

Considering the 2019 guideline [11], one could conclude that aCT de-escalation, which was already increasingly implemented in daily practice throughout 2013–2016 despite the guideline (“guideline update anticipation”), has now been translated into official guidelines. Next to age and receptor-status, aCT-indication is primarily based on grade, tumor size, and the degree of lymph-node involvement [11]. In addition, there remains a role for the 70-GS as a supportive tool, however, it is important to realize that also the 70-GS-indication itself is guided by grade, histologic subtype, tumor size, N-status and receptor-status [11].

Bearing the clinical implications of grading in mind, it is clear that accurate grading by pathologists is of utmost importance to provide high-quality care. In that respect, we have previously shown that there is substantial variation in grading between Dutch pathology laboratories [15]. For example, > 50% of Dutch pathology laboratories showed significantly deviant ORs in a multivariate logistic regression model, in which the average laboratory was considered the reference [15]. However, from these data, we cannot conclude which laboratories may grade inaccurately. Nonetheless, considering the fact that grade is the determinative factor in > 10,000 patients, while > 50% of Dutch laboratories graded significantly different from the nationwide average, it may be clear that for many thousands of IBC-patients, treatment may have been based on inaccurate grading.

This underlines that maximum effort to minimize differences in grading is urgently required. Therefore, we launched two encouraging initiatives, i.e. laboratory-specific feedback reports and training of pathologists by e-learning [27, 28]. This is an ongoing process, yet, even after training and feedback, grading variation remains considerable. Therefore, additional avenues should be explored, such as the potential supportive role of artificial intelligence in analyzing histopathology data [29, 30]. In addition, Ki67 is a prognostic proliferation marker that may be of added value, but there is controversy with regard to its clinical utility in routine management due to variation in analytical practice, lack of a standardized procedure for Ki67-assessment, and absence of consensus on cutoff-values [31, 32].

Although this study applies to the Dutch situation, we would like to emphasize that breast cancer guidelines in the Netherlands are generally in accordance with international guidelines [5–7, 9, 10, 12, 33, 34], which all take grade into account (eTable 4). While for some guidelines primary focus has shifted to GEP-use as a first determinant in deciding whether aCT should be administered [7, 10, 33], these guidelines also state that, when GEPs are not available, classic clinicopathologic biomarkers, including grade should be considered. In this light, it is highly relevant to underline that GEPs are not accessible (i.e. $4.000 per 21-GS, $3.900 per 70-GS) nor applicable for every IBC patient [35–42]. For example, probably only up to half of the eligible women in the US receive GEP-testing [40, 41]. Hence, for the majority of patients, both in developing and developed countries, classic pathology biomarkers remain the most important indicator(s) for the need of adjuvant systemic treatment. Moreover, recent evidence shows that, in specific subgroups, the 21-GS score may be accurately predicted based on grade and PR-receptor status, perhaps saving the need for expensive tests [43–47].

The short follow-up time precluded outcome analyses. Thus, whether aCT de-escalation in almost 40% of IBC patients with a strict guideline aCT-indication did not compromise their outcome remains to be elucidated. This important analysis will be performed after longer follow-up.

A limitation of this study is the exclusion of patients who received neoadjuvant treatment. As neoadjuvant systemic therapy is the preferred initial approach in HER2-driven and TNBC [7], these groups are most likely underrepresented in our study. Hence, we would like to emphasize that the 30,843 IBC patients within our study do not represent the overall population of IBC patients in the Netherlands. However, they do represent the nationwide population of IBC patients who were potentially eligible for adjuvant systemic therapy between 2013 and 2016.

### Conclusions

This study with nationwide data on 30,843 Dutch primary breast cancer patients illustrates the increasing importance of histologic grading with regard to tailoring adjuvant systemic therapy, as the indications for aCT and aET depend on grade in roughly a third of IBC patients. This is due to the large proportion of patients who fall into the category in which histologic grade finally determines the need for adjuvant therapy when national guidelines are followed. This stresses the need for optimizing grading by pathologists, to diminish the risk of worse patient outcome due to non-optimal treatment decisions.

## Supplementary Information

Below is the link to the electronic supplementary material.

Supplementary Information 1 (DOCX 71 kb)

## Data Availability

Data are available upon request from PALGA (the nationwide registry of histo- and cytopathology in the Netherlands) and the Netherlands Cancer Registry (NCR).
