# Supplementary material for: The increasing importance of histologic grading in tailoring adjuvant systemic therapy in 30,843 breast cancer patients
Source: Breast Cancer Res Treat. 2021 Jan 30;187(2):577–86. doi: 10.1007/s10549-021-06098-7 (PMC8189961; doi:10.1007/s10549-021-06098-7)
Supplement: Supplementary file 1 — Supplementary Information 1 (DOCX 71 kb) [file 10549_2021_6098_MOESM1_ESM.docx]

**Supplementary files**

eFigure 1. Flowchart of included patients with invasive breast cancer to assess the role of histologic grading in tailoring adjuvant systemic therapy in the Netherlands

| **eTable 1.** Number of breast cancer patients for whom the indication for adjuvant chemotherapy (aCT) depends on histologic grade according to the Dutch breast cancer guideline between 2013 and 2016 | |
| --- | --- |
| **Patient- and tumor characteristics** | **Number of patients** |
| All patients aged <35 years, with a N0 tumor ≤1cm* | 48 |
| All patients aged 35-70 years, with a N0 tumor between 1·1-2cm* | 7,696 |
| Total | **7,744 (25.1%)** |
| * grade I: no indication for chemotherapy, grade II-III: indication for chemotherapy | |

| **eTable 2.** Number of breast cancer patients from our dataset (2013-2016) for whom the indication for adjuvant chemotherapy (aCT) would depend on histologic grade according to the current Dutch breast cancer guideline (2019) | |
| --- | --- |
| **Patient- and tumor characteristics** | **Number of patients with an indication for aCT solely based on grade** |
| Age <35 years, HER2 negative, N0/N0(i+)/N1(mi), tumor 1·1-2cm* | 84 |
| Age 35-70 years, ER/PR positive, HER2 negative, N1-3, tumor ≤2cm* | 1,790 |
| Age 35-70 years, ER/PR positive, HER2 negative, N0/N0(i+)/N1(mi), tumor 1·1-3cm** | 8,995 |
| Total number of patients (n=30.843) | **10,869 (35.2%)** |
| * chemotherapy for grade II-III tumors ** tumor 1·1-2cm: chemotherapy in case of grade III, tumor 2·1-3cm: chemotherapy in case of grade II-III | |

| **eTable 3.** Number of breast cancer patients from our dataset (2013-2016) for whom the indication for adjuvant endocrine therapy (aET) would depend on histologic grade according to the current Dutch breast cancer guideline (2019) | |
| --- | --- |
| **Patient- and tumor characteristics** | **Number of patients with an indication for aET based on grade** |
| All ages, ER and/or PR positive (≥10% of the tumor cells show ER- and/or PR-specific staining on IHC),N0/N0(i+)/N1(mi), tumor 1·1-2cm | 9,173 |
| Total number of patients (n=30,843) | **9,173 (29.7%)** |
| * endocrine therapy for grade II-III tumors | |
|  | |

| **eTable 4.** Indication for adjuvant chemotherapy (aCT) in early breast cancer according to the different international guidelines |
| --- |
| **Specific (inter)national guidelines** |
| **Cancer Care Ontario (CCO) guideline [12]** |
| - High risk N0 tumor |
| - Tumor >0.5cm with ≥1 of the following risk-factors |
| - **Grade III** |
| - Triple negative (ER-, PR-, HER2-) |
| - Lymphovascular invasion |
| - HER2 + receptor status |
| - Oncotype DX recurrence score associated with an estimated 10-year relapse risk of ≥15% |
| - Adjuvant!Online risk >10-15% |
| **American Society of Clinical Oncology guideline (ASCO) (CCO guideline endorsement) [5, 6, 9]** |
| - High risk N0 tumor (exception: tubular and mucinous carcinomas: possibly no aCT) |
| - Tumor >0.·5cm with ≥1 of the following risk-factors |
| - **Grade III*** |
| - Triple negative (ER-, PR-, HER2-) |
| - Lymphovascular invasion* |
| - HER2 + receptor status |
| - Oncotype DX recurrence score associated with an estimated 10-year relapse risk of >20% |
| - Adjuvant!Online risk >10-15% |
| **National Comprehensive Cancer Network (NCCN) guideline [10]** |
| - Ductal, lobular, mixed, metaplastic; pT1, pT2 or pT3; pN0; >0.5cm |
| - Strongly consider Oncotype DX |
| - aCT <70 years for Recurrence score 26-30 or ≥31 |
| - No Oncotype DX |
| - Take into account factors like N-status, tumor size, **tumor grade**, lymphovascular invasion, age, comorbid conditions |
| **European Society for Medical Oncology (ESMO) [36]** |
| - Luminal A-like (ER+, HER2-, Ki67 low, PR high, low-risk molecular signature (if available)) |
| - High disease burden (≥4LN, ≥T3) |
| - Luminal B-like (HER2-) (ER+, HER2- and either Ki67 high or PR low, high-risk molecular signature (if available)) |
| - Majority of cases aCT, dependent on individual risk score, expected response to aET and patient preference |
| - Luminal B-like (HER2+) (ER+, HER2+, Ki67, any PR) |
| - All patients |
| **St. Gallen Expert Panel 2019 [7]** |
| - Genomic signatures preferred for basing the critical yes/no chemotherapy decision |
| - If genomic signatures are not available, integrate traditional pathology (tumor size, grade, ER/PR and proliferation) to recommend aCT or not |
| * Grade 3 and presence of lymphovascular should generally not be used to drive decision making when considered in isolation and must be interpreted in the overall clinical context, also: no chemo for well differentiated luminal A like tumors |
